# Attention to space and time: Independent or interactive systems? A narrative review

**DOI:** 10.3758/s13423-023-02325-y

**Published:** 2023-07-05

**Authors:** Mariagrazia Capizzi, Ana B. Chica, Juan Lupiáñez, Pom Charras

**Affiliations:** 1 Mind, Brain and Behavior Research Center (CIMCYC), Department of Experimental Psychology, University of Granada, Granada, Spain; 2 Univ Paul Valéry Montpellier 3, EPSYLON EA 4556, F34000 Montpellier, France

**Keywords:** Spatial orienting, Temporal orienting, Rhythm, Endogenous, Exogenous, SOA distributions

## Abstract

While there is ample evidence for the ability to selectively attend to *where* in space and *when* in time a relevant event might occur, it remains poorly understood whether spatial and temporal attention operate independently or interactively to optimize behavior. To elucidate this important issue, we provide a narrative review of the literature investigating the relationship between the two. The studies were organized based on the attentional manipulation employed (endogenous vs. exogenous) and the type of task (detection vs. discrimination). Although the reviewed findings depict a complex scenario, three aspects appear particularly important in promoting independent or interactive effects of spatial and temporal attention: task demands, attentional manipulation, and their combination. Overall, the present review provides key insights into the relationship between spatial and temporal attention and identifies some critical gaps that need to be addressed by future research.

## Introduction

Adaptation to our dynamic environment requires the ability to selectively attend to *where* in space and *when* in time a relevant event might occur. Although a link between spatial and temporal attention can be easily envisaged in many everyday scenarios, from safely crossing a busy intersection to quickly reacting to a falling object, spatial and temporal attention have often been investigated separately. On an applied level, elucidating whether spatial and temporal attention optimize behavior either independently or interactively is, however, critical in several situations requiring both types of attention (e.g., driving and aircraft flying settings). On a theoretical level, a better understanding of the interplay between different forms of attention could inform future neurocognitive and computational models of human attention. A prominent hypothesis in this regard is that temporal attention would depend on the synchronization of neuronal activity spatially based on receptive fields representing the attended location (i.e., spatiotemporal view; Doherty et al., 2005; Nobre & van Ede, 2018; Rohenkohl et al., 2014). According to this view attending to *when* is contingent on knowing *where* an event will occur; yet, there is also evidence that temporal attention may occur independently of spatial attention (e.g., MacKay & Juola, 2007; Tal-Perry & Yuval-Greenberg, 2022; Weinbach et al., 2015). Therefore, identifying the conditions that promote independent or interactive effects of spatial and temporal attention can also contribute to the debate on whether temporal attention operates in a spatially or nonspatially specific manner.

As detailed below, there are different ways to deploy attention to space and time (Capizzi & Correa, 2018; Chica et al., 2013; Seibold et al., 2023). Such heterogeneity is also reflected at the conceptual level with similar terms often referring to different theoretical constructs and task manipulations. This results in a complex picture of the relationship between spatial and temporal attention. Our goal is to address this complexity in a narrative review of the studies investigating both spatial and temporal attention. We begin with a summary of the main assumptions and terminology commonly used in the spatial and temporal literatures, to provide a useful background for the subsequent description of studies combining the two types of attention (note that spatial and temporal attention studies have been summarized separately in previous reviews; e.g., Chica et al., 2014, and Nobre & van Ede, 2018, respectively). We then define the conceptual framework and the criteria followed to organize the selected studies. Since the objective of this review is to elucidate whether spatial and temporal attention operate independently or interactively to enhance behavior, we have exclusively included studies that investigated their relationship. Studies that solely compared spatial and temporal attention separately (e.g., Griffin, Miniussi & Nobre, 2002; Sharp, Melcher, & Hickey, 2019; Tang et al., 2013) or that focused on dimensions other than the spatial and temporal ones (e.g., social orienting, Hayward & Ristic, 2015; memory processes; Jones et al., 2022) have been excluded. In the Discussion section, we summarize the main findings regarding the relationship between spatial and temporal attention and highlight some critical gaps that require to be addressed by future research.

## Spatial attention

**Fig. 1:**
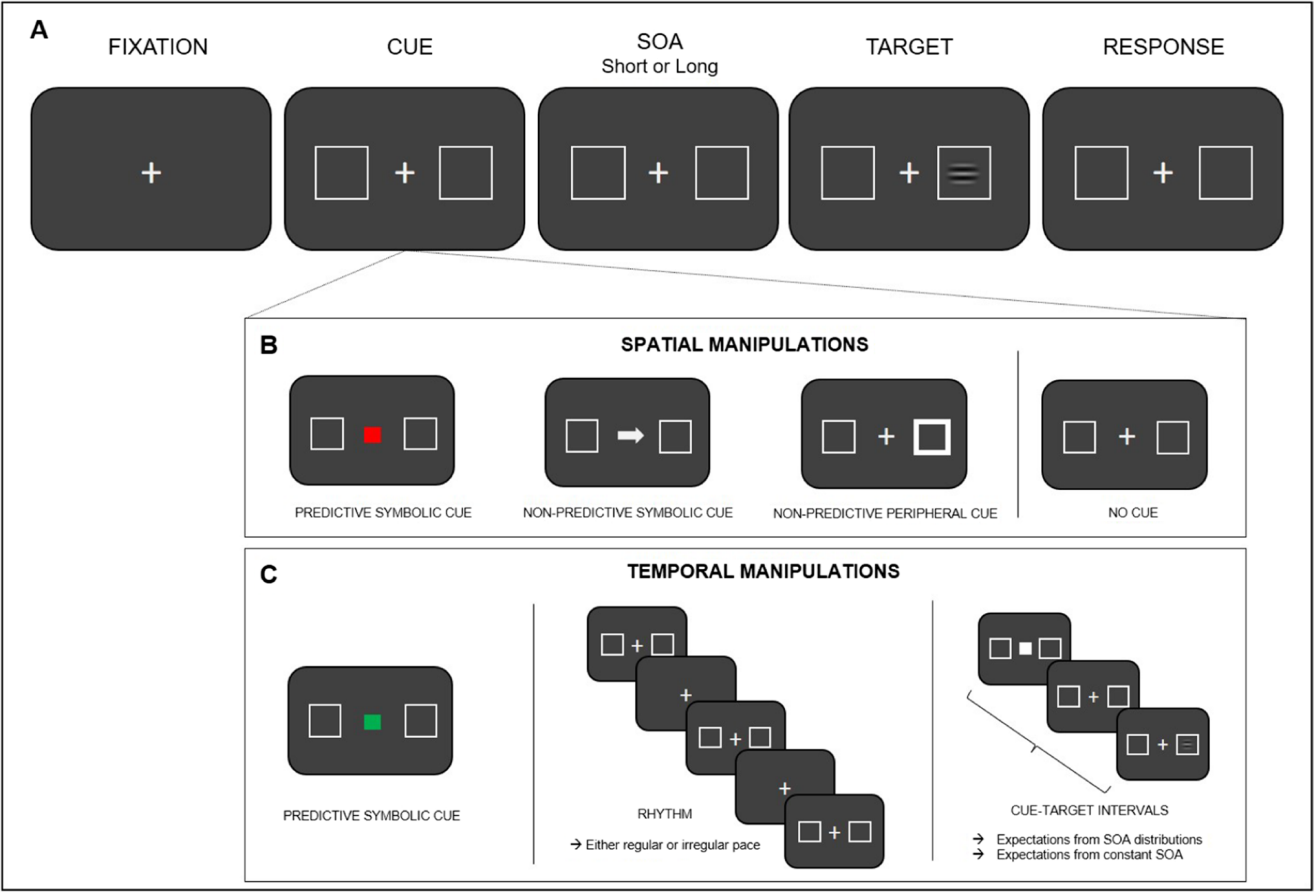
**(A)** Schematic representation of a typical spatial/temporal orienting trial comprising, in this example, a fixation cross, a cue, two placeholders (one located to the left and one to the right side of the fixation cross), and a target stimulus. The cue and the target are separated by either a short or a long Stimulus Onset Asynchrony (SOA). **(B)** The first three displays show examples of the most commonly used spatial cues to direct participants’ attention to a particular target location (the right side in this example). The predictive symbolic cue (left-sided image) is represented by a red (or green) square that signals with high probability (e.g., 75% validity) that the target is most likely to appear on one side of the screen (color meaning counterbalanced across participants). The central arrow (middle image) is an example of non-predictive symbolic cues (50% validity) pointing to the right location (please refer to the section entitled Combining spatial and temporal attention for a more detailed explanation of this type of cue). The change in luminance of the right placeholder (right-sided image) illustrates a non-predictive peripheral cue attracting exogenous attention to the right location (50% validity). The fourth image in the row depicts that spatial attention may be also deployed without cues, such as, for example, when instructing participants to orient attention to the left or the right side of space within a block (see section entitled Spatial attention). **(C)** Schematic overview of the most commonly used temporal attention manipulations. In a temporal variant of the spatial orienting task (left image), a predictive symbolic cue (a green or a red square) orients participants’ attention (e.g., 75% validity) to either the short or long SOA (color meaning counterbalanced across participants). Temporal expectations can be also exogenously driven by rhythmic structures that, in this example (middle image), are created by repeating the placeholders either at a regular or irregular pace. Another way of orienting attention to time is to manipulate the duration of the cue-target interval either between or within blocks (right image; see section entitled Temporal attention). Please note that spatial attention may be also deployed with other tasks, such as visual search tasks, reviewed in the text but not represented here (see Rolke et al., 2016, for an illustrative example of visual search tasks)

Visuospatial attention has been widely investigated using the spatial orienting task (i.e., “cost and benefits paradigm”; Posner, 1980; Posner et al., 1978). The original version of the spatial orienting task comprised the following stimuli: a fixation cross, a cue, two placeholders (one lateralized to the left and one to the right side of the fixation cross), and a target (Fig. 1A). Extending this initial design, variants of the spatial orienting task may employ more than two placeholders or no placeholders at all to explore attentional orienting to target locations (reviewed in Chica et al., 2014). The spatial cue and the target are separated by a time interval known as Stimulus Onset Asynchrony (SOA). The way in which cue type and SOA are manipulated during the spatial orienting task depends on the researcher's interest in "endogenous" versus "exogenous" attentional modes (Jonides, 1981).

The term *endogenous* (or top-down) refers to the ability to orient attention according to our goals and external task demands. To investigate endogenous attention in the spatial orienting task, cue type and cue validity are manipulated as follows. The cue consists of a centrally displayed symbol that predicts with high probability the spatial location of the target. For instance, a red square may indicate that the target is most likely to appear on the right side of space (e.g., 75% cue validity), whereas a green square may be associated with the left side (color meaning counterbalanced across participants; Fig. 1B). Trials can be categorized as valid (or cued) or invalid (or uncued) based on whether the target appears at the location indicated by the cue or at the non-indicated location, respectively. Otherwise, trials are defined as neutral when cues have no spatial meaning and the target is equally likely to appear at all possible spatial locations (50% cue validity). The presence of a neutral condition allows distinguishing between attentional “benefits,” which refer to shorter reaction times (RTs) and/or higher accuracy for valid as compared to neutral trials, and attentional “costs” referring to longer RTs and/or lower accuracy for invalid as compared to neutral trials (Posner, 1980).

Endogenous spatial cues may be arbitrary, like in the above color example, or they may have a spatial meaning such as when using arrows. Because of the daily exposure to arrow stimuli, arrows can nonetheless orient attention to space even when they do not predict the target location (50% cue validity; Callejas, Shulman, & Corbetta, 2014; Hein et al., 2006; Lasaponara, et al., 2011). In this case, arrow cues engage a distinct form of involuntary attentional orienting (i.e., “automated symbolic orienting”), which has been shown to operate independently from endogenous and exogenous spatial attention (Ristic & Kingstone, 2012). Especially for arbitrary cues, participants are usually informed of the cue-target contingencies employed during the task and encouraged to use the spatial cues to predict the target location. Under some specific manipulations of the spatial orienting task, trial-by-trial learning of cue-target contingencies may be implicit, with participants inferring across trials that a given target location is more probable than another location (Vossel et al., 2014; see also O’Reilly et al., 2013). For completeness, it should be noted that endogenous spatial attention can be also elicited without cues, for example, by instructing participants about which side they have to attend to within a given block (e.g., Marzecová et al., 2018; Sani et al., 2021; Seibold et al., 2020). In all of these cases, the canonical finding is that RTs become faster and/or accuracy is higher for valid compared to invalid trials, the so-called “validity effects,” which usually last for several seconds in both detection and discrimination tasks (Chica et al., 2014). In addition to cue type and cue validity, endogenous spatial attention requires long SOAs (~ 300 ms) to enable decoding of the cue meaning.

In contrast to endogenous spatial attention, *exogenous* (or bottom-up) spatial attention refers to the automatic and irrepressible deployment of attention toward a salient and potentially relevant location. To induce exogenous attention during the spatial orienting task, the cue is generally manipulated peripherally rather than centrally. An example of exogenous spatial cues is the change in luminance of one of the two placeholders located on the left and right sides of space. Due to its saliency, the peripheral change will automatically attract participants’ attention to that location. Although peripheral cues are typically non-predictive of the target location (i.e., the target appears at the cued location on 50% of the trials and at the uncued location on the remaining 50%), participants’ RTs and/or accuracy are better on cued as compared to uncued trials, provided, however, that short SOAs (~ 100 ms) are employed. At longer SOAs (~ 300 ms, depending on the task at hand; Lupiáñez et al., 1997), exogenous validity effects reverse, with better performance on the uncued compared to the cued location, an effect labeled Inhibition of Return (IOR). As its name suggests, IOR was originally attributed to an inhibition of the return of attention to a previously attended location (Posner et al., 1985). More recently, alternative motor and perceptual accounts have been put forward, which respectively explain IOR either as a reluctance to respond to (Taylor & Klein, 2000) or as a cost in detecting a new stimulus presented in a previously attended location (Lupiáñez et al., 2013). In summary, behavioral evidence for endogenous and exogenous attention in the spatial orienting task can be observed by manipulating cue type (central vs. peripheral), cue predictiveness (predictive vs. non-predictive), and SOA duration between the cue and the target.

Although until now we have only introduced the spatial orienting task because of its greater use in the studies reviewed here, there are also other tasks to examine visuospatial attention such as visual search tasks (Wolfe, 2021). In visual search tasks, participants are required to detect the presence or absence of a given target among distractors. Thus, in addition to spatial attention (*where*), visual search tasks also tap feature-based attention (*what*). In the present review, only two studies employed a visual search task to investigate the relationship between spatial and temporal attention (Rolke et al., 2016; Seibold et al., 2020).

## Temporal attention

Research in temporal attention focuses on the study of “temporal expectations in attention,” which refers to the ability to anticipate when a relevant stimulus will occur to optimize its processing at the perceptual and response level. Hence, in a broader sense, the terms *attention* and *expectation* are often used interchangeably in this literature (Nobre & van Ede, 2018).

Temporal expectations can be established in different ways (Fig. 1C). A temporal variant of the spatial orienting task allows testing for the ability to endogenously attend to the likely timing of target appearance (Coull & Nobre, 1998; Kingstone, 1992). In a typical temporal orienting task, a centrally displayed symbol (e.g., a green square) may predict with high probability (e.g., 75% cue validity) that the target will appear after a short SOA,^1^ whereas a red square might be associated with a long SOA (color meaning counterbalanced across participants). Participants are typically instructed to use the temporal cues to anticipate the moment when the target will occur. Echoing the spatial validity effects, participants benefit from advanced temporal information as indexed by shorter RTs and/or higher accuracy when the target appears at the expected vs. unexpected SOA. However, differently from the spatial orienting task, temporal validity effects are usually restricted to the short SOAs only, whereas the effects are reduced or even absent at the long SOAs (Capizzi, Correa, & Sanabria, 2013; Coull & Nobre, 1998; Griffin et al., 2002; Miniussi et al., 1999). This is due to the fact that once a target that was cued to appear at the early SOA fails to materialize participants will re-orient their attention to the long SOA, as the probability of target occurrence increases with time. The benefit afforded by elapsing time is formally described by the hazard function, which represents the probability that an event will occur given that it has not occurred yet (Coull, 2009; Herbst et al., 2018; Janssen & Shadlen, 2005; Niemi & Näätänen, 1981; Visalli et al., 2019; see below for further details). Evidence supporting the existence of a re-orienting process on “delayed” invalid trials (cf. Coull, 2011) comes from the finding of significant temporal orienting effects at long SOAs when catch trials (i.e., trials without target presentation) are introduced in the temporal orienting task (Correa et al., 2006b). Catch trials discourage re-orienting of attention to the long SOA by inducing uncertainty about target appearance (Drazin, 1961; Näätänen, 1972). However, it is important to consider that not only catch trials impact temporal expectations regarding target occurrence but also response preparation, leading to a general RT slowing especially pronounced during long SOA trials (Capizzi et al., 2015; Correa et al., 2004).

This brief overview of the temporal orienting task highlights that while temporal expectations can be driven endogenously by symbolic cues, similar to the spatial orienting task, they are also influenced by the passage of time itself. Even without explicit temporal cues, it is still possible to predict the likely moment of target onset based on the SOA probability distribution. To illustrate this point, consider a simple RT task in which the target is separated from a neutral warning signal by three possible SOAs.^2^ In the most common “aging” distribution, all SOAs have the same a priori probability of being presented (.33, in this example). According to the hazard function, if the target does not occur at the short SOA, the conditional (a posteriori) probability that it will occur at the medium SOA raises to .5, and if it does not occur at the medium SOA, the conditional probability of occurrence at the long SOA will equal 1. Participants’ RTs follow the hazard function, with faster responses as the SOA increases (Langner et al., 2018; Niemi & Näätänen, 1981; Vallesi et al., 2013).

The finding of faster responses at longer SOAs in an aging distribution shows that participants’ temporal expectations increase as time elapses during the trial. This certainty can be eliminated by making the cue uninformative of when the target will occur. In the so-called “non-aging” distribution, the conditional probability of target occurrence is maintained constant throughout the three SOAs by increasing the a priori probability of target presentation at the short SOA (i.e., 4/8 in the short SOA; 2/8 in the medium SOA; 1/8 in the long SOA; and 1/8 in catch trials). A non-aging distribution makes the target equally likely to appear (or not to appear) at all SOAs (Gabay & Henik, 2008). By contrast, if the a priori probability of the long, rather than the short, SOA increases, as occurs in an “accelerating aging” distribution, RTs become faster with longer SOAs due to an accelerated conditional probability of target appearance at the long SOA (Trillenberg et al., 2000).

Within the framework of the above SOA literature, it should be finally acknowledged that temporal attention may also be manipulated using a “constant SOA design,” which consists of keeping one SOA interval fixed for the entire block and varying it between blocks (e.g., Bausenhart et al., 2008). When comparing performance on “short” and “long” SOA blocks, the common finding is that participants are faster in the shorter as compared to the longer blocks (Mattes & Ulrich, 1997). This advantage has been interpreted as evidence that temporal attention to target onset is enhanced by a short SOA interval as compared to a longer and more variable one (Rolke et al., 2016). However, another likely explanation for the finding of faster RTs during the short SOA block is that time estimation becomes less precise with longer interval durations (i.e., Weber’s law; Gibbon, 1977), which makes it difficult to completely disentangle the contribution of temporal attention and time-estimation processes to the benefits conferred by a constant short SOA interval (Seibold et al., 2020).

In addition to endogenous temporal cues and SOA manipulations, temporal expectations can be exogenously driven by regular rhythmic structures. According to the Dynamic Attending Theory (Large & Jones, 1999), natural brain rhythms are conceived of as endogenous oscillations that can be entrained by external rhythms. The repetition of a regular rhythmic pattern is thought to synchronize participants' oscillations with improved accuracy and/or response speed when the target occurs in time with the rhythm, as compared to a target occurring out of time (earlier or later), or when the target occurs after a rhythmic as compared to an arrhythmic sequence (Cravo et al., 2013; de la Rosa et al., 2012; Jones et al., 2002; Mathewson et al., 2010; Rohenkohl et al., 2012; Sanabria et al., 2011). Unlike the temporal orienting effects described above, rhythms can automatically orient attention to specific moments in time. For instance, participants can benefit from rhythms even when explicitly instructed to ignore those (Rohenkohl et al., 2011), or when the rhythm actually hampers task performance (Breska & Deouell, 2014). It is, then, assumed that rhythm-based temporal expectations are established in a purely exogenous fashion, although some studies have also employed rhythms as predictive symbolic cues (Doherty et al., 2005; Triviño et al., 2011). An example of predictive rhythms can be found in the Discrimination tasks section.

In summary, there is more than one way to develop temporal expectations about when a target might occur and more than one task to study temporal attention. A common characteristic among all these tasks is the absence of spatial uncertainty regarding the upcoming target location being cues and targets almost always presented in the center. It should nonetheless be noted that the presence of spatial certainty in the deployment of temporal attention has been mostly neglected in studies of temporal attention (e.g., Korolczuk et al., 2018). We come back to this issue in the General discussion section. The following paragraph provides a general overview of how the reviewed studies were organized based on the theoretical and methodological issues briefly presented up to this point.

## Combining spatial and temporal attention 

Although spatial attention is traditionally divided into endogenous and exogenous orienting modes, some attentional effects cannot be fully understood within this dichotomy. For instance, arrow cues may orient attention to the pointed location even when they are not predictive of where the target will appear (Ristic & Kingstone, 2012), or the effects elicited by a recent history of spatial attentional deployments can be unrelated to the participant's current goals and physical salience of the stimuli (see Awh et al., 2012, for a review of visual search tasks).

Similar to spatial attention, a rigid endogenous-exogenous dichotomy in temporal attention may not fully capture the nature of the processes underlying different sources of temporal expectation. As an example, the term endogenous (as well as related terms like “voluntary” or “volitional”) have been often used for task designs in which the SOA probability distribution of target onset changes between participants (hereafter referred to as “mixed designs”; e.g., Gabay & Henik, 2008; Milliken et al., 2003). Because participants are uninformed about the specific SOA probability distribution, temporal expectations gradually build up through experience with the probabilistic structure of the task, thus differing from the endogenous temporal expectations elicited by symbolic cues on a trial-by-trial basis (e.g., Capizzi et al., 2012; Duma, Granziol, & Mento, 2020; Nobre, 2010; Tal-Perry & Yuval-Greenberg, 2022; Visalli et al., 2021, 2023).

**Fig. 2:**
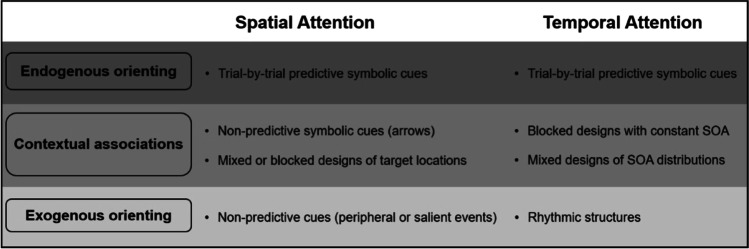
Schematic overview of the various attentional manipulations used in the reviewed studies. Please note that these manipulations can be combined in several ways to investigate specific combinations of spatial and temporal attention. Examples include endogenous spatial and endogenous temporal attention, or endogenous spatial attention combined with temporal attention based on contextual associations. For more detailed information on these combinations and their specific findings, please refer to the main text

Based on the above considerations, it becomes apparent that manipulations of spatial and temporal attention cannot always be classified into a strict endogenous-exogenous dichotomy, an issue that adds further complexity when combining these two attention types within the same experimental protocol. To account for this aspect, our review embraced a nuanced view of endogenous and exogenous orienting manipulations (Fig. 2). The most endogenous form of attentional manipulations includes trial-by-trial orienting of attention to space and/or time through the use of predictive symbolic cues (i.e., mixing valid vs. invalid cues or 100% valid vs. neutral cues). Orienting of attention based on symbolic cues necessitates interpretation of the cue on each trial and the proactive use of cue information to facilitate performance on the subsequent expected target. The most exogenous form of attentional manipulations involves the automatic orienting of attention driven by the characteristics of the stimulus itself rather than goal-driven intentions. In the context of spatial attention, exogenous orienting is deployed by salient non-predictive cues, whereas exogenous temporal attention is elicited by rhythmic structures that are typically task-irrelevant for the participant.

The progression from endogenous to exogenous attentional manipulations involves a mixture of both top-down and bottom-up processes. The key feature of this “middle category” is that orienting of attention is not established on a trial-by-trial basis through predictive cues, but rather it occurs through more automatic associations that arise from exposure to environmental contingencies (such as in the case of arrows) or from experience with a specific task context. Concerning arrows, we consider here the case of non-predictive arrow cues (i.e., 50% cue validity) that may still orient attention to the target location even without the involvement of endogenous attentional control (Ristic & Kingstone, 2012). As part of task context manipulations, we include mixed and constant (blocked) designs, which are collectively referred to as “contextual associations.” As mentioned above, in a mixed design, the SOA probability distribution of target onset (or target location) varies across blocks and/or participants. On the other hand, in a constant design, a single SOA interval (i.e., either short or long) or a single target location (i.e., left or right) is presented throughout the block.^3^ Another example of a constant design is one in which a specific task instruction is utilized in a blocked manner to selectively orient participants’ attention during a block of trials (e.g., attend exclusively to the left side of space during one block of trials and to the right side in a different block of trials). In both mixed and constant manipulations, after gaining initial experience with the task structure, the orienting of spatial or temporal attention can occur through contextual associations. Moreover, once these contextual associations are established, participants can maintain a more constant attentional setting compared to a trial-by-trial design, thus, lowering the reliance on endogenous control.

In addition to the endogenous-exogenous dimension, the type of task to be performed was also considered. Indeed, while detection tasks have been mainly associated with independent effects of spatial and temporal attention (e.g., Tal-Perry & Yuval-Greenberg, 2022; Weinbach et al., 2015), discrimination tasks have been shown to favor the interaction between spatial and temporal attention (Doherty et al., 2005; Rohenkohl et al., 2014). The type of task to be performed might then help elucidate the conditions under which independent or interactive effects of spatial and temporal attention are observed. The majority of the reviewed studies employed behavioral methods, with some of them also reporting brain measures from functional magnetic resonance imaging (fMRI) or electrophysiological (M/EEG) recording. When appropriate, the behavioral findings are complemented with the neural ones derived from these methods.

## Trial-by-trial orienting of endogenous spatial and endogenous temporal attention

The studies reviewed in this section employed predictive symbolic cues to orient endogenous attention to space and time. As there are no studies targeting both detection and discrimination requirements in the same protocol, detection and discrimination tasks are presented separately.

### Detection tasks

A total of four studies combined endogenous spatial and endogenous temporal attention using simple detection tasks.

The seminal study by Coull and Nobre (1998) was the first to compare the neural bases of spatial and temporal attention triggered by predictive symbolic cues. Positron emission tomography (PET) and fMRI data were acquired while participants were administered with spatial, temporal, and combined spatiotemporal orienting conditions. For spatial orienting, an arrow-like cue (created by the brightening of one side of a centrally displayed diamond) indicated whether to attend to the left or the right side of space with 80% validity. In the temporal orienting condition, the brightening of an inner (or an outer) circle signaled that the target was most likely to appear after a short (or a long) SOA (300 vs. 1,500 ms, respectively). In the combined condition, the brightening of one of the circles and one side of the diamond indicated one of the four possible spatiotemporal combinations (i.e., left-short, right-short, left-long, and right-long). For the neutral condition, the diamond and the circles brightened at once, thus providing neither spatial nor temporal information. The participant's task was to detect the onset of a target stimulus as quickly as possible. At the behavioral level, significant validity effects of spatial, temporal, and combined spatiotemporal cues (as compared to neutral cues) were reported. Brain imaging data showed a partial overlap in the neural areas mediating spatial and temporal orienting alongside a clear hemispheric asymmetry, with preferential involvement of the right parietal cortex for spatial orienting, and the left parietal cortex for temporal orienting. Moreover, the parietal cortex was activated bilaterally in the combined spatiotemporal condition.

Although Coull and Nobre (1998) provided evidence for a behavioral benefit of their spatiotemporal cues, the cues never conveyed discordant information about each other (e.g., a valid temporal cue but an invalid spatial one). This makes it difficult to fully assess the additive or interactive nature of the relationship between spatial and temporal attention. The same issue applies to a subsequent study by Olk (2014), in which the symbolic cues (arrow-like symbols for spatial orienting and circles for temporal orienting) used by Coull and Nobre (1998) were compared with purely symbolic cues (i.e., line-width cues for both spatial and temporal orienting; 80% cue validity). The most important result of Olk’s (2014) study was that, when using the same cues as Coull and Nobre, the spatiotemporal effects were larger than the temporal effects alone, and they also tended to be larger than the spatial effects. By contrast, with purely symbolic line-width cues, the spatiotemporal cues did not add any further advantage to that already conferred by spatial or temporal cues alone. These findings were interpreted as evidence that the deployment of spatiotemporal attention might differ according to the symbolic nature of the used spatial and temporal cues.

Olk’s (2014) study also manipulated spatial and temporal validity together such that the question of how spatial and temporal attention would behave when the validity rate is independently manipulated remained unanswered. A step toward addressing this question was made by Weinbach et al. (2015). They presented participants with separate spatial, temporal, and spatiotemporal orienting tasks. Cues consisted of symbolic central cues (shapes or colors) that either predicted the spatial location or the moment of target onset (400 ms or 1,400 ms). In the combined spatiotemporal condition (always presented after the spatial and temporal blocks), the cue was a colored shape obtained from the mixture of the previously used color and shape cues. The key feature of Weinbach et al. (2015) study was the orthogonal manipulation of cue validity (75%) in the combined spatiotemporal task. Results showed that spatial and temporal validity effects did not interact; spatial validity effects were significant irrespective of whether the target occurred at the expected or unexpected SOA. Similarly, temporal validity effects were significant at both attended and unattended spatial locations.

A further EEG study manipulated spatial and temporal attention in the auditory modality (Faugeras & Naccache, 2016). Four types of bilateral auditory cues were intermixed across separate blocks: fully predictive cues (100%) of either location (left or right ear) or onset (1,000 or 2,000 ms) of a monaurally presented target; predictive cues of both target location and onset, and non-predictive cues. Behaviorally, the effects of spatial and temporal attention did not interact and were, respectively, reflected in the modulation of the cue-locked P3 and the contingent negative variation (CNV) amplitudes.

In summary, the main message of the above-reviewed studies is that endogenous spatial and temporal attention can operate independently in the context of simple detection tasks.

### Discrimination tasks

The four studies reviewed below combined endogenous spatial and endogenous temporal attention using discrimination tasks.

In the EEG study by Doherty et al. (2005), a ball proceeded from the left to the right side of the screen before disappearing under an occluding band. Upon its reappearance, participants had to discriminate a ball with a dot versus a ball without a dot and to make a fast response only to the ball with the dot (i.e., go/no-go task). Spatial attention was built up by the spatial trajectory of the ball as it moved across the screen prior to going behind the occluding band, which could be either predictable or unpredictable, whereas the regular or irregular pace at which the ball moved was used to set up temporal attention. The orthogonal crossing of the ball’s trajectory and pace also gave rise to combined spatiotemporal (predictable trajectory and regular pace) and neutral (unpredictable trajectory and irregular pace) conditions. In contrast to the work reviewed above, the deployment of spatial and temporal attention was hence generated through visual rhythmic cues rather than a single symbolic cue. However, the explicit nature of the instructions given to participants (i.e., attending to the rhythms to anticipate target appearance) and the predictive nature of the rhythmic information (100% valid) differentiate the study by Doherty et al. (2005) from the exogenous rhythm studies reviewed below. The behavioral results showed significant main effects of spatial and temporal attention with no interaction between the two attention types. At the EEG level, spatial attention, but not temporal attention, modulated the P1 amplitude. Yet, there was a significant spatial by temporal attention interaction, such that the P1 amplitude was larger in the spatiotemporal than in the spatial condition alone. These EEG findings were interpreted by Doherty et al. (2005) as evidence that temporal attention optimized visual perceptual processing only in conjunction with spatial attention. The lack of a behavioral interaction was attributed to the task demands, which were not high enough to observe the same interactive pattern at the level of behavior (an issue addressed in a follow-up study by the same group as reviewed below; Rohenkohl et al., 2014). Indeed, it could be argued that the go/no-go task employed by Doherty et al. (2005) stressed detection more than discrimination demands (see also the Discussion section).

An auditory analog of the visual task used by Doherty et al. (2005) was devised by Rimmele et al. (2011). In this EEG auditory version, the trajectory of a moving sound (either regular or irregular in space and time) was perceived as traveling from the right to the left (or from the left to the right) across the top of the participant’s head, generating spatial, temporal, combined spatiotemporal, or no expectation about target reappearance from an occluding white noise band. A complex tone was the target (presented on 50% of the trials; go trials), whereas a pure tone was the non-target (50% of no-go trials). Similar to the original study by Doherty et al. (2005), participants were explicitly instructed to rely on the information provided by the trajectory of the moving sound on valid trials (100% validity) to anticipate the target. Behaviorally, significant validity effects were found for the temporal, but not the spatial, condition that alone did not confer any benefit to behavior and did not interact with temporal expectations. The EEG data showed that temporal attention modulated early auditory (P1 and N1 potentials) and task-related processing stages (N2 potential), whereas spatial attention affected later processing stages (P3 potential) only when combined with temporal attention, as evidenced by a significant interaction between spatial and temporal attention. These findings confirm that temporal processing plays a more important role than spatial processing in the auditory modality and that the effects of combined spatial and temporal attention on target anticipation, as measured by event-related potentials (ERPs), differ for auditory and visual task demands. Regardless of these domain-related differences, most germane to the present review is the finding of independent effects of spatial and temporal attention at the behavioral level and of interactive effects at the EEG level.

A further study combining endogenous spatial and endogenous temporal attention in a discrimination task was conducted by MacKay and Juola (2007). Participants performed a rapid serial visual presentation (RSVP) task that required searching for one of two target letters. The spatial location of the targets in the stream and/or their temporal lags of appearance could be predicted (or not in neutral trials) by fully valid (100%) color cues in a block-wise fashion. Target identification in the RSVP stream was enhanced by both spatial and temporal cues, whose effects combined additively with no interaction between them (i.e., the benefits for spatiotemporal cues were similar to the combination of separate spatial and temporal benefits).

The study by Rohenkohl et al. (2014) is the only one of those reviewed in this section reporting a behavioral interaction between endogenous spatial and endogenous temporal attention in a discrimination task. A central colored arrow predicted both where (arrow direction) and when (arrow color) a peripheral Gabor would appear (80% validity). The participant’s task was to discriminate the orientation of the Gabor. Temporal expectations alone did not improve perceptual discrimination but temporal cues benefitted performance only at spatially attended locations, as evidenced by a significant interaction between spatial and temporal attention.

Together, the studies manipulating endogenous spatial and temporal attention in discrimination tasks provide a more fragmented picture as compared to the studies using detection tasks. Indeed, interactive effects of spatial and temporal attention were observed at the behavioral level only when task demands were perceptually high (Rohenkohl et al., 2014), or just emerged at the EEG level (Doherty et al., 2005; Rimmele et al., 2011).

### Spatial attention combined with temporal attention based on contextual associations

In the studies reviewed in this section, temporal expectations were triggered by contextual associations within either an endogenous or an exogenous spatial orienting task. We begin with the studies combining endogenous spatial attention and temporal contextual associations.

Girardi and colleagues (2013) tested to what extent spatial attention driven by predictive symbolic cues was sensitive to the probability of target occurrence in both detection and discrimination tasks. Participants were presented with arrow cues (80% valid) and divided into three groups as a function of the probability of target onset at one of three SOAs (300, 500, or 800 ms). For each group, the target was more likely to appear at one SOA, with the remaining trials equally divided among the two other SOA conditions (i.e., for the middle SOA group, the target appeared at the 500-ms SOA in 66% of the trials, and at the 300- and 800-ms SOAs in 17% of the trials). For both detection and discrimination tasks, spatial validity effects were significant only at the more probable SOA, whereas at the less probable SOAs (no matter whether short or long) they were not reliable.

A very recent study by Tal-Perry and Yuval-Greenberg (2022) further investigated the relationship between endogenous spatial attention and contextual temporal expectations, but with a different goal to Girardi and colleagues (2013). Tal-Perry and Yuval-Greenberg sought to elucidate whether temporal attention based on contextual information was independent or not from endogenous spatial attention. A symbolic arrow-like cue was presented on each trial to validly (50%), invalidly (25%), or neutrally (25%) signal the location of the target in a detection task. The SOA between the spatial cue and the target could assume one among five values (500, 900, 1,300, 1,700, 2,100 ms), with the key manipulation that the SOA distributions (i.e., uniform/20% probability for each SOA vs. inverse-U-shaped/a ratio of 1:2:3:2:1 among the five SOAs) varied between participants. Results showed significant spatial RT benefits (i.e., shorter RTs for valid vs. neutral trials) and costs (i.e., longer RTs for invalid vs. neutral trials) as well as the classic effects of SOA duration (i.e., shorter RTs at longer SOAs) and SOA distributions (i.e., linear and quadratic effects for the uniform and inverse-U-shaped distribution, respectively), with no significant interaction between spatial and temporal attention.

In the context of exogenous spatial attention, previous research examined whether participants could benefit from the implicit temporal information provided by longer SOAs between the peripheral spatial cue and the target in an aging distribution. To this aim, such studies changed the predictability of target onset by introducing different SOA probability distributions^4^ (Gabay & Henik 2008, 2010; Milliken et al., 2003). Of note, when using an exogenous spatial orienting task with short and long SOAs, it is common to observe validity effects (i.e., RT decrease on cued vs. uncued trials) at shorter SOAs, but IOR (i.e., RT increase on cued vs. uncued trials) at longer SOAs. Since different mechanisms underlie validity effects at short SOAs and IOR at long SOAs (Danziger et al., 1998; Enns & Richards, 1997; Tassinari et al., 1994; Tassinari & Berlucchi, 1993), in the scope of the present review, our focus in the Discussion section is only on validity effects at short SOAs. However, we provide a brief mention of IOR findings in this section, as the primary goal of the below studies was to investigate the modulation of IOR by temporal attention rather than validity effects.

Milliken et al. (2003) manipulated the probability that a target would appear after a peripheral spatial cue using three SOA (100/500/900 ms) conditions: an unbiased condition (i.e., an equal proportion of trials at each SOA, that is, aging distribution), a short-biased condition (i.e., a higher proportion of trials at the 100-ms SOA), and a long-biased condition (i.e., a higher proportion of trials at the 900-ms SOA). In one experiment, participants performed a detection task, whereas, in a second experiment, they performed a discrimination task. Spatial and temporal attention did not interact in the detection task, but they interacted in the discrimination task, with larger validity effects at short SOAs and enhanced IOR for the short-biased condition as compared to the two other conditions.

Gabay and Henik (2008; 2010) also altered the temporal predictability of a peripheral spatial cue by manipulating the probability of occurrence of four possible SOAs (100, 400, 700, or 1,000 ms) within a common aging distribution (i.e., increased probability of target appearance across SOAs), a non-aging distribution (i.e., equal probability of target appearance at all SOAs), and an accelerated aging distribution (i.e., increased probability of target appearance at the longest SOA). Results showed that for the simple detection task (Gabay & Henik, 2008), neither the validity effects at the short SOAs nor IOR at the long SOAs were modulated by temporal attention. By contrast, for the discrimination task (Gabay & Henik, 2010), the validity effects at the short SOAs were only observed for the aging and non-aging distribution, but not for the accelerated aging distribution, the one conveying the highest temporal information. Moreover, IOR was abolished in the non-aging distribution, whereas it appeared earlier in the accelerated aging distribution as compared to the aging distribution.

Taking into account the studies reviewed in this section, except for Giradi and colleagues (2013), it seems that spatial attention driven by either endogenous or exogenous cues and temporal expectations based on contextual associations work independently in simple detection tasks. For discrimination tasks, temporal expectations may instead shape the time course of spatial validity (and IOR) effects either increasing or abolishing them.

### Temporal attention combined with spatial attention based on contextual associations

The studies reviewed below combined temporal attention, deployed by either contextual associations or predictive symbolic cues, with spatial contextual associations.

Linking back to our definition of environmental associations, the study by Hayward and Ristic (2016) used spatially non-predictive arrows (50% cue validity) but temporally predictive symbolic cues (i.e., the brightening of a large or a small circle) to signal the likely moment of target onset (100 or 1,200 ms; 88% cue validity). The task was a simple detection task. Participants were presented with blocks comprising spatial trials, temporal trials, combined spatiotemporal trials, and no-cue trials. Spatial and temporal cues were orthogonally manipulated in the combined spatiotemporal trials. Results showed significant validity effects for both spatial and temporal cues. When spatial and temporal cues were presented together, spatial and temporal validity effects did not interact, thus, confirming the finding of independent effects of endogenous spatial attention (with predictive arrow cues) and endogenous temporal attention in detection tasks (Weinbach et al., 2015).

Rolke et al. (2016) investigated the relationship between spatial and temporal attention by pairing a visual search task with EEG. Spatial attention was manipulated through instructions; participants had to attend to one side of the search display (e.g., the left side) and to discriminate whether the upper or lower part of a target stimulus (i.e., a salient singleton of a given shape and color embedded among homogenous distractors) was missing only if the target appeared on the attended, but not unattended, spatial location (e.g., the right side in this example). Temporal attention was manipulated by keeping a constant short or long SOA (600 or 2,200 ms) between the offset of an auditory warning signal and the beginning of the search display during the entire block. Participants were explicitly required to use the warning signal as a temporal reference to estimate the onset of the search display. RTs to targets were reduced in the short SOA blocks, as anticipated in this type of SOA design, but temporal attention did not interact with spatial (or feature-based) attention to enhance visual processing (note also that no significant main effect of temporal attention nor significant temporal by spatial attention interactions were found in other behavioral measures such as accuracy in go trials and false alarm rates on no-go trials). The EEG results showed distinctive ERP modulations by spatial and temporal attention (i.e., greater amplitudes of the N2pc, SPCN, and P3 potentials for spatial attention, while enhanced N1 amplitude and shorter N2pc latency for temporal attention), with no interaction between the two attention types across all levels of stimulus processing. Based on the EEG data, Rolke et al. (2016) concluded that temporal attention does not rely on spatial (and feature-based) attention to aid visual processing in a visual search task. However, findings from a follow-up EEG study by the same group (Seibold et al., 2020) threw support for this conclusion into question.

The two studies differed only in the way temporal expectations were provided, by a constant SOA duration in Rolke et al. (2016) and by symbolic cues in Seibold et al. (2020). In the latter study, a high or low pitch tone predicted (75% cue validity) on a trial-by-trial basis one of the two SOAs (600 or 2,200 ms) at which the search display was likely to appear. Differently from Rolke et al. (2016), Seibold et al.’s (2020) study reported a significant interaction between spatial and temporal attention at the EEG level, such that the effects of spatial (and feature-based) attention during early visual processing (indexed by the N1) were observed only in the temporal valid, but not invalid, condition (of note, the temporal by spatial attention interaction was not analyzed behaviorally). The main conclusion of the two described EEG studies was that temporal and spatial attention work interactively to enhance visual processing in a visual search task only when temporal expectations are driven by symbolic cues but not when a constant SOA manipulation is used.

Unlike the studies reviewed so far, Beck et al. (2014) manipulated spatial and temporal attention in a continuous performance task in which no symbolic cues were presented but instead targets followed one another after an inter-stimulus interval (ISI). Across separate blocks, the target could always appear at the same location, at one of two possible locations, or at one of four possible locations. Likewise, the ISI between targets could be either fixed (always 1,250 ms) or could increase from two (1,000 and 1,500 ms) to four different values (500, 1,000, 1,500, and 2,000 ms). This design created three levels of fully crossed spatial and temporal attention conditions (high, medium, and low). In Experiment 1, participants performed a simple detection task, whereas in Experiment 2 they had to discriminate between two target colors. Significant main effects of spatial and temporal attention were found in both experiments; however, the two attention types interacted only in the detection, but not the discrimination, task. Specifically, while RTs decreased as temporal expectations increased (from low to high) across all spatial attention conditions, spatial RTs were significantly shorter in the high temporal condition than in the medium and low ones. In other words, the presence of high spatial and high temporal expectations led to faster RTs. It should be noted, however, that this interactive pattern could also be explained by a more precise response synchronization strategy in the condition with only one possible target location and one single ISI. Indeed, the interaction between spatial and temporal attention vanished when response preparation was prevented for by discrimination requirements.

In summary, the heterogeneous nature of the three last studies reviewed in this section (Beck et al., 2014; Rolke et al., 2016; Seibold et al., 2020) makes it difficult to draw a conclusion on the relationship between spatial and temporal attention in visual search and sustained attention tasks.

### Spatial attention combined with rhythm-based temporal attention 

The relationship between spatial attention and rhythm-based temporal attention has just begun to be explored. In three out of the five reviewed studies, spatial attention was deployed by endogenous cues in detection (Jones, 2015; Kizuk & Mathewson, 2017) and discrimination tasks (Wilsch et al., 2020), one study used an exogenous spatial manipulation in a discrimination task (Ahrens et al., 2015), whereas another one focused on endogenous and exogenous spatial attention using both detection and discrimination tasks (Jones, 2019).

Jones (2015) presented participants with a spatial orienting task comprising either symbolic visual or auditory cues (i.e., four X letters surrounding the fixation cross and high- or low-frequency tones), which were repeated four or five times to create a rhythm sequence. The color of the visual cues or the frequency of the tones indicated whether to attend to the left or right side of space (75% cue validity). Conversely, the rhythm had no bearing on the task to be completed, and participants were instructed not to pay attention to it. The target (either visual or auditory) could appear in-time or out-of-time with the preceding rhythm (i.e., earlier or later). In separate conditions, the modality of the rhythmic cues and the target could match (e.g., both visual) or mismatch (e.g., one visual and the other auditory). The main result of Jones’ (2015) study was that endogenous spatial attention and rhythm-based temporal attention did not interact in either unimodal or cross-modal conditions. It is, however, important to note that although entrainment models (e.g., Large & Jones, 1999) would predict faster responses for in-time than out-of-time (early and late) targets, there were instead no significant differences between in-time and late targets. This finding could be accounted for by the hazard function, that is, the increasing conditional probability that the target will appear given that it has not yet occurred (Herbst et al., 2018; Janssen & Shadlen, 2005; Visalli et al., 2019). Thus, both entrainment effects driven by rhythms and SOA effects implicitly driven by the passage of time contributed to participants’ RTs in Jones’ (2015) study.

In the EEG study by Kizuk and Mathewson (2017), participants performed a spatiotemporal task in which a symbolic cue predicted the upcoming target location (70% cue validity). The cue and the target were spaced by eight visual stimuli that were presented bilaterally (above the horizontal meridian) as entrainers of alpha oscillations (i.e., 12-Hz rhythmic presentation). After the onset of the last entrainer, a backward masked target was displayed on the attended or unattended side of space, either in-time or out-of-time with the rhythmic stimuli. Participants had to indicate the side (left or right) that the target appeared. At the behavioral level, there was a significant interaction between spatial and temporal attention such that accuracy was higher for in-time as compared to out-of-time targets in the spatially unattended location only, whereas differences were smaller and not significant in the spatially attended condition. The behavioral benefits for in-time targets were also associated with both increased alpha power and increased neural entrainment of the alpha phase in the hemisphere processing the unattended locations.

A recent magnetoencephalographic (MEG) study (Wilsch et al., 2020) combined endogenous spatial attention and rhythm-based temporal expectations in cross-modal auditory and visual discrimination tasks (i.e., visual cues were paired with auditory targets, whereas auditory cues with visual targets). In the visual task, spatial attention was manipulated with valid (100% cue validity) or neutral arrow cues, pointing to either the left or right ear or to both ears, respectively. In the auditory task, a tone to the left or right ear indicated the left or right hemifield, whereas a tone to both ears provided no spatial information. Temporal expectations were established by repeating the visual or auditory cue four times in a rhythmic sequence. The targets (visual: a “T” or an inverted “T” character; auditory: a sound increasing or decreasing in pitch) could occur in phase (within one to four cycles) after the rhythmic cues (80% of trials), or antiphase (20% of trials) with respect to the rhythm. In a separate random condition, the cue was presented continuously for the same total duration as the rhythmic cue with the target occurring at a random time point after the cue offset. Besides an expected stronger effect of spatial attention in the visual domain, and of temporal expectations in the auditory domain, there was no significant main effect of temporal expectations on behavior (i.e., no difference between rhythmic vs. random trials), but only a small performance improvement for in phase versus antiphase trials. However, spatial and temporal expectations interacted such that spatial validity effects on RTs (i.e., valid vs. neutral trials) were stronger for the random condition than for the rhythmic condition. At the neural level, the rhythmic cue increased post-cue synchronization of low-frequency delta (1–3 Hz) oscillations especially for the auditory task, whereas for spatial attention a typical pattern of alpha lateralization in the visual system was observed.

Regarding the combination of exogenous spatial attention and rhythm-based temporal expectations, the study by Ahrens et al. (2015) implemented a visual pre-target motion paradigm similar to the above-mentioned one by Doherty et al. (2005). Participants were presented with a matrix of 5 × 9 circles. The circles located in the row below the fixation cross flashed successively from left to right or from right to left, at a rhythmic or arrhythmic pace, creating an apparent motion effect that ended with the central circle. Targets, consisting of an “x” or “+” symbol requiring a discrimination response, could be presented in one of the left or right circles adjacent to the central one (i.e., in- or out-of-motion). The motion of the circles was task-irrelevant by instructions and, importantly, non-predictive of either target position or timing of target appearance (i.e., 50% of the targets occurred in- or out-of-motion, at a rhythmic or arrhythmic pace). In addition to explicit instructions to ignore motion information, the authors implemented an endogenous (orthogonal) spatial manipulation using symbolic cues (i.e., valid or neutral cues with 75% and 50% cue validity, respectively). The reasoning was to further make the motion stimuli task-irrelevant, by providing participants with endogenous cues that indicated at the beginning of each trial the location of the upcoming target. This design resulted in significant benefits for endogenous spatial cues (i.e., higher accuracy and faster RTs for validly cued targets), which were, however, independent of those afforded by implicit spatial and temporal motion, the main focus of the study. This finding was interpreted as evidence that participants did not endogenously process the motion stimuli. More importantly, results showed that the spatial and temporal benefits exogenously conferred by apparent motion were independent.

Unlike the previously reviewed studies, which all employed visual and/or auditory stimuli, the last reviewed study explored the relationship between spatial and temporal attention in the tactile modality (Jones, 2019). Across two experiments, a series of four or five tactile taps forming a rhythm were used as spatial cues. In Experiment 1, comprising detection (i.e., a single tap) and discrimination tasks (i.e., a single tap vs. a double tap), the rhythmic cue was presented bilaterally and indicated whether to attend to the left or right hand (75% cue validity). In Experiment 2, which included a detection task only, the rhythmic cue was unilateral and non-predictive of the upcoming target location (either left or right hand). In both experiments, the target could appear in- or out-of-time with the preceding rhythm that, by instructions, was task-irrelevant. As in his previous study (Jones, 2015), this rhythmic manipulation resulted in a combination of entrainment and SOA effects. Indeed, participants were equally fast for in-time and late targets, thus supporting an account based on the hazard function. In Experiment 1, this temporal advantage was independent of spatial attention and was the same for both detection and discrimination tasks. Conversely, in Experiment 2, the exogenous spatial manipulation resulted in a significant IOR, with faster RTs for uncued (i.e., the target appeared at a different hand than the one receiving the rhythm) than cued targets. IOR interacted with the preceding rhythm such that, for the cued condition only, it increased for in-time as compared to out-of-time targets.

In summary, studies combining spatial attention and rhythm-based temporal expectations show either independent or interactive effects that appear to be contingent not only on the type of task to be performed but also on whether spatial attention is endogenously or exogenously manipulated.

## Discussion

The main goal of the present review was to provide a qualitative summary of the studies combining spatial and temporal attention to shed light on their independent or interactive relationship. Table 1 summarizes the main findings.

**Table 1:** Summary of the main findings of the reviewed studies based on the attentional manipulations detailed in Fig. 2, type of task, and perceptual demands. Asterisks indicate studies in which independent effects of spatial and temporal attention were observed at the behavioral level, while interactive effects at the EEG level. The presence of double asterisks indicates that only EEG findings were available from the study

Main findings	Spatial attention	Temporal attention	Task	Perceptual demands	Study
Independent	Endogenous orienting (Predictive symbolic cues)	Endogenous orienting (Predictive symbolic cues)	Detection	Low	Coull & Nobre (1998)
					Faugeras & Naccache (2016)
					Olk (2014)
					Weinbach et al. (2015)
			Discrimination	Low	Doherty et al. (2005)*
					MacKay & Juola (2007)
					Rimmele et al. (2011)*
		Contextual associations (SOA distributions)	Detection	Low	Tal Perry & Yuval-Greenberg (2022)
		Rhythms	Detection	Low	Jones (2015)
					Jones (2019)-Experiment 1
			Discrimination	Low	Jones (2019)-Experiment 1
	Contextual associations (Non-predictive arrow cues)	Endogenous orienting (Predictive symbolic cues)	Detection	Low	Hayward & Ristic (2016)
	Contextual associations (Mixed design)	Contextual associations (Mixed design)	Discrimination	Low	Beck et al. (2014)
	Contextual associations (Blocked instructions)	Contextual associations (Constant SOA design)	Discrimination	Low	Rolke et al. (2016)
	Exogenous orienting (Non-predictive cues)	Contextual associations (SOA distributions)	Detection	Low	Gabay & Henik (2008)
					Milliken et al. (2003)
		Rhythms	Discrimination	Low	Ahrens et al. (2015)
Interactive	Endogenous orienting (Predictive symbolic cues)	Endogenous orienting (Predictive symbolic cues)	Discrimination	High	Rohenkohl et al. (2014)
		Contextual Associations (SOA distributions)	Detection	Low	Girardi et al. (2013)
			Discrimination	Low	Girardi et al. (2013)
		Rhythms	Detection	High	Kizuk & Mathewson (2017)
			Discrimination	High	Wilsch et al. (2020)
	Contextual associations (Blocked instructions)	Endogenous orienting (Predictive symbolic cues)	Discrimination	Low	Seibold et al. (2020)**
	Exogenous orienting (Non predictive cues)	Contextual Associations (SOA distributions)	Discrimination	Low	Gabay & Henik (2010)
					Milliken et al. (2003)
		Rhythms	Detection	Low	Jones (2019)-Experiment 2

Despite the great heterogeneity between studies, the relationship between spatial and temporal attention seems to be contingent on three interrelated factors: the type of task to be performed, the attentional manipulation employed, and the combination of the two. The role of task in the relationship between spatial and temporal attention is not entirely surprising as already highlighted by prior work. Specifically, independent effects of spatial and temporal attention have been mostly related to detection tasks (e.g., Weinbach et al., 2015), whereas interactive effects to discrimination tasks (e.g., Doherty et al., 2005; Rohenkohl et al., 2014). However, a wider examination of the literature covered here shows that the type of task alone is not always a key factor influencing the relationship between spatial and temporal attention (see Table 1). Indeed, we reviewed the evidence for independent effects of spatial and temporal attention in discrimination tasks (e.g., MacKay & Juola 2007) and for interactive effects in detection tasks (e.g., Beck et al., 2014). It is, thus, important to reconsider the role of task from a broader perspective by further delving into the manipulation of spatial and temporal attention.

Much of the reviewed studies relied on trial-by-trial manipulations of endogenous spatial and temporal attention. When the task was a simple RT task, studies (Coull & Nobre, 1998; Faugeras & Naccache, 2016; Olk, 2014; Weinbach et al., 2015) did all converge on independent effects of endogenous spatial and endogenous temporal attention regardless of the modality (visual or auditory) or types of cues used (arrows, colors, or shapes). This conclusion also found support in the only study combining endogenous temporal cues with non-predictive arrow cues (considered here as an example of spatial environmental associations) in a detection task (Hayward & Ristic, 2016). When the task involved discrimination, the relationship between endogenous spatial and temporal attention showed a mixed pattern of effects, with both independent and interactive effects observed (Doherty et al., 2005; MacKay & Juola 2007; Rimmele et al., 2011; Rohenkohl et al., 2014).

Narrowing down the focus on the above findings, we would argue that it is important to examine to what extent the discrimination task stressed perceptual demands. Indeed, while independent effects of endogenous spatial and temporal attention were observed in simple (and go/no-go) discrimination tasks entailing the selection of a specific response according to target features (Doherty et al., 2005; MacKay & Juola 2007; Rimmele et al., 2011), interactive effects were found when the discrimination task involved a deeper perceptual analysis of the target (Rohenkohl et al., 2014). It is, thus, possible that the behavioral benefits conferred by endogenous spatial and endogenous temporal attention to simple discrimination tasks would partly depend on motor preparation of the (two) possible responses, a process that could be optimized by both attention types separately as in simple RT tasks (Correa et al., 2005; Nobre, 2010). By contrast, endogenous temporal attention boosted target processing only at valid spatial locations in more demanding perceptual tasks (i.e., Gabor discrimination task; Rohenkohl et al., 2014). Interestingly, interactive patterns of endogenous spatial and endogenous temporal attention also occurred at the EEG, but not at the behavioral level, in go/no-go discrimination tasks requiring response inhibition (Doherty et al., 2005; Rimmele et al., 2011). Behavioral measures were perhaps limited in their ability to capture the interaction between endogenous spatial and temporal attention in go/no-go tasks that still tapped motor more than perceptual processes. Overall, the review of the studies manipulating endogenous spatial and endogenous temporal attention supports and clarifies the task-dependent nature of independent and interactive effects, with the former occurring in detection and discrimination tasks with minimal perceptual demands and the latter emerging in discrimination tasks with higher perceptual demands. In the remainder of the discussion, we scrutinize whether this picture also holds for other attentional manipulations, starting with the studies combining spatial attention and temporal attention based on contextual associations.

The relationship between spatial and temporal attention also appears to be task-dependent when combining exogenous spatial attention and temporal expectations driven by contextual associations (i.e., manipulation of SOA probability distributions), with some differences, however, as compared to the endogenous temporal manipulations discussed above. The studies reviewed in this category (Gabay & Henik, 2008; 2010; Milliken et al., 2003) showed independent effects of spatial and temporal attention for detection tasks, while interactive effects were observed for discrimination tasks, even if the latter only imposed minimal perceptual demands. By contrast, a consistent pattern failed to emerge for manipulations of endogenous spatial attention and temporal expectations driven by contextual associations. Girardi and colleagues (2013) reported that spatial validity effects were reliable only when the target occurred at the more probable SOA in a given probability distribution for both simple detection and discrimination tasks, whereas Tal-Perry and Yuval-Greenberg (2022) showed that the effects of endogenous spatial attention and temporal expectations based on contextual associations were independent in a simple RT task. This discrepancy could have been driven by several methodological factors that could be explored more directly in future research. Overall, as a preliminary conclusion, exogenous and endogenous spatial attention appear to operate independently of temporal expectations based on contextual associations in detection tasks (except for Girardi et al., 2013).

The studies combining spatial attention triggered by contextual associations with temporal attention provide mixed evidence regarding their underlying relationship. In two studies where spatial attention was set up through instructions in a blocked-wise manner, spatial and temporal attention exerted independent effects when temporal expectations were deployed by a constant SOA duration (Rolke et al., 2016), but interactive effects when they were established by trial-by-trial symbolic cues (Seibold et al., 2020). Because these studies diverged only in the way temporal information was provided, they suggest that manipulation of temporal attention, but not task demands, was responsible for the interactive effects of spatial and temporal attention. The study by Beck et al. (2014) further supports the role of attentional manipulation on spatiotemporal outcomes by reporting differential effects in discrimination and detection tasks when target onset and target location were inferred from inter-trial relationships (rather than overt cues). Together, findings from these studies point to the difficulty of deriving a single message that relies exclusively on task demands when spatial attention is triggered by contextual associations.

Additional evidence for a complex interplay between attentional modulation and type of task comes from the studies combining rhythm-based temporal expectations and spatial attention, summarized below. Jones (2015) found that endogenous spatial attention and rhythm-based temporal expectations were independent in unimodal (visual and auditory) and cross-modal (visual-auditory and auditory-visual) detection tasks. A subsequent study by the same author (Jones, 2019, Experiment 1) extended these findings to the tactile modality by showing independent effects of endogenous spatial attention and rhythm-based temporal expectations in both simple detection and discrimination tasks. By contrast, Kizuk and Mathewson (2017) and Wilsch et al. (2020) observed an interactive pattern between endogenous spatial attention and rhythms-based temporal expectations. In the former study, temporal benefits were stronger for the unattended spatial location, whereas in the latter study, spatial benefits were stronger for the random temporal condition. It is worth noting that despite the different nature of these interactions, both studies used a perceptually demanding task. The task by Kizuk and Mathewson (2017) required the detection of a backward-masked target, whose luminance was individually adjusted in a staircase procedure. Detection of a masked target is particularly challenging for the perceptual system as it involves the ability to discriminate the target from the mask. In Wilsch et al. (2020) study, participants had to discriminate between (individually adjusted) complex sounds in the auditory condition and complex visually presented stimuli in the visual condition.

As mentioned above for endogenous manipulations, the divergent findings of independent effects for endogenous spatial attention and rhythm-based temporal expectations in discrimination tasks (Jones, 2019, Experiment 1), and interactive effects in detection tasks (Kizuk & Mathewson, 2017) can be explained by considering the perceptual demands of the task, with low and high perceptual demands favoring independent and interactive effects, respectively. Although plausible, one might object that this claim contradicts some of the findings reviewed here showing interactive, instead of independent, effects of spatial and temporal attention in detection and discrimination tasks with minimal perceptual demands (Beck et al., 2014; Gabay & Henik, 2008; 2010; Girardi et al., 2013; Milliken et al., 2003). We rather believe that, as a whole, these divergent findings reinforce the idea that a better understanding of the relationship between spatial and temporal attention cannot prescind from a deeper analysis of both attentional manipulation and perceptual task demands that go beyond a simplistic distinction between detection and discrimination requirements.

Further considering the significance of perceptual task demands in the relationship between spatial and temporal attention, it is interesting that one of the most debated questions in the early stages of temporal attention research revolved around whether temporal attention could enhance perceptual processes or not (Correa et al., 2005, 2006a; Nobre, 2010). For a long time, the general idea was that, unlike spatial attention, the benefits of temporal attention were constrained to motor processes, with more research nowadays challenging this view by reporting robust temporal attention effects on perceptual processes (e.g., Breska & Ivry, 2021; Davranche et al., 2011; Rohenkohl et al., 2012; Vangkilde et al., 2012). However, in the majority of temporal attention studies there is usually no spatial uncertainty as to where the target will occur, such that it is still possible that perceptual processing could be a key determinant in the relationship between spatial and temporal attention (at least for some of the manipulations outlined here). This topic deserves further investigation.

Regarding exogenous spatial attention and rhythm-based temporal expectations, the two types of attention operated independently in a simple discrimination task (Ahrens et al., 2015), while interactively in a detection task (Jones, 2019, Experiment 2). Nonetheless, it should be noted that the interaction reported in Jones (2019) concerned IOR instead of validity effects. As different mechanisms underlie IOR and validity effects (Danziger et al., 1998; Enns & Richards, 1997; Lupiáñez et al., 2013), more studies combining exogenous spatial and rhythmic temporal attention are warranted to draw meaningful conclusions on their underlying relationship. The combination of exogenous spatial and exogenous temporal attention clearly deserves high priority in the future given the paucity of existing studies.

The following aspects should be finally acknowledged. Although the main aim of this review was not to verify whether temporal attention operates in a spatially or nonspatially specific manner (Doherty et al., 2005; Rohenkohl et al., 2014; Nobre & van Ede, 2018), it still provided some useful insight into this issue. According to the studies reviewed here, the evidence for a spatially specific nature of temporal attention was limited to the combination of endogenous spatial and endogenous temporal attention within a perceptually demanding task (Rohenkohl et al., 2014; see also Doherty et al., 2005). In contrast to the spatially specific nature of temporal attention, there was evidence for the following patterns: (1) temporal expectations driven by contextual associations developed independently of spatial attention (Tal-Perry & Yuval-Greenberg, 2022); (2) rhythm-based temporal expectations showed benefits at unattended, rather than attended, spatial locations when combined with endogenous spatial attention in a perceptually demanding task (Kizuk & Mathewson, 2017); and (3) rhythm-based temporal expectations and endogenous spatial attention operated independently in simple detection and discrimination tasks (Jones, 2015; 2019). Moreover, there was evidence that spatial attention could be contingent on temporal attention to boost visual processing in visual search tasks (Seibold et al., 2020). It should be further noted that most of the research reviewed here has been on vision, where space is dominant, and almost always using tasks stressing spatial requirements (e.g., detection or discrimination of stimuli presented in the left or right visual field). As a final remark, it is important to acknowledge that additional methodological factors can impact the relationship between spatial and temporal attention. These factors include the symbolic nature of the spatiotemporal cues (Olk, 2014), the use of static or moving cues to endogenously orient attention to space and/or time (Doherty et al., 2005), or the specific SOA probability distribution employed in a block (Tal-Perry & Yuval-Greenberg, 2022). Coupled with task demands and attentional manipulations, these methodological aspects should be carefully considered when combining spatial and temporal attention in a single paradigm (see also Seibold et al., 2023). It is also worth mentioning that once more data from studies of spatial and temporal attention become available, quantitative meta-analysis could assist in resolving some of the inconsistencies observed in the literature.

Overall, the present review provides a thorough synthesis of the studies investigating both spatial and temporal attention. As mentioned in the Introduction, understanding the relationship between spatial and temporal attention is critical from theoretical and practical perspectives. Theoretical models of selective attention have made significant progress in formalizing how endogenous attention is deployed over space (e.g., Ni & Maunsell, 2019) or time (e.g., Denison et al., 2021). However, further efforts should be devoted to integrating the spatial and temporal dimensions and, as shown in this review, to gaining a better understanding (and future modeling) of exogenous manipulations. Future research on the combination of spatial and temporal attention could also be applied to enhance driving and aircraft safety, for example, by devising effective strategies to improve the integration of attention to relevant spatial locations and optimal moments in time.

## Conclusion

Based on the reviewed studies, the following issues should be carefully considered and/or addressed in future work:

1. A refinement of the processes underlying spatial and temporal attention, and of the used terminology, is necessary to build up a sound framework for the studies combining spatial and temporal attention.
2. More parametric manipulations of task demands for spatial and temporal attention should be done in the same individuals to better clarify the role of task demands in the relationship between the two attention types.
3. More auditory, tactile, and cross-modal studies are warranted to explore to what extent findings from the visual modality can be generalized to other modalities in which time, instead of space, is the dominant dimension. Moreover, tasks stressing temporal requirements over spatial ones are highly encouraged.
4. More studies with manipulations different to endogenous ones are needed to draw meaningful conclusions on other, yet under-represented, attentional manipulations (i.e., very few studies of exogenous spatial and exogenous temporal attention, or other combinations of interest).

Hopefully, the findings from this review will inspire further investigations into the combined effects of spatial and temporal attention. Since the ability to orient attention to space and time is fundamental to our interaction with the environment, theoretical advancements could finally be translated into practical research.

## Data Availability

Data sharing is not applicable to this article as new data were not created or analysed during this review.

## Footnotes

1 Please note that in the temporal literature, the term *foreperiod* is more commonly used than SOA to indicate the time interval between the warning signal/cue and the target. However, for the sake of compatibility with the spatial literature, the term SOA refers here to both spatial and temporal cue-target intervals.

2 One might wonder whether temporal expectations overlap with general alerting processes since the presentation of a warning signal/cue before the target also changes the state of participants’ alertness speeding up RTs with respect to a no-cue condition (Posner & Petersen, 1990). Although we acknowledge the methodological similarity between manipulations of alerting and temporal expectations in warned RT tasks, it should be noted that temporal expectations are functionally different and independent from general alerting effects (McCormick et al., 2018; Weinbach & Henik, 2012). Studies on the relationship between alerting and spatial attention are hence not covered here.

3 Although a blocked design with a constant SOA interval could have an inherent rhythmic structure, this is different from the exogenous rhythmic manipulations reviewed here. In a constant SOA manipulation, the rhythmic part is indeed common to both short and long SOA conditions, and “rhythm” is not the key variable under consideration. By contrast, in an exogenous rhythmic task the key comparison is between regular versus irregular rhythmic sequences, or between targets occurring in-phase as compared to targets occurring out-of-phase with respect to a preceding rhythm.

4 Another way to manipulate the temporal predictability of a peripheral spatial cue is to introduce a large proportion of catch-trials (Laidlaw & Kingstone, 2017; Tipper & Kingstone, 2005). However, as mentioned earlier, catch-trials also affect response preparation processes in addition to temporal expectations. These studies are thus not covered here.

## References

[CR1] Ahrens M-M, Veniero D, Gross J, Harvey M, Thut G (2015). Visual benefits in apparent motion displays: automatically driven spatial and temporal anticipation are partially dissociated. PLoS ONE.

[CR2] Awh E, Belopolsky AV, Theeuwes J (2012). Top-down versus bottom-up attentional control: a failed theoretical dichotomy. Trends in cognitive sciences.

[CR3] Bausenhart KM, Rolke B, Ulrich R (2008). Temporal preparation improves temporal resolution: evidence from constant foreperiods. Perception & Psychophysics.

[CR4] Beck MR, Hong SL, van Lamsweerde AE, Ericson JM (2014). The effects of incidentally learned temporal and spatial predictability on response times and visual fixations during target detection and discrimination. PLoS ONE.

[CR5] Breska A, Deouell LY (2014). Automatic bias of temporal expectations following temporally regular input independently of high-level temporal expectation. Journal of Cognitive Neuroscience.

[CR6] Breska A, Ivry RB (2018). Double dissociation of single-interval and rhythmic temporal prediction in cerebellar degeneration and Parkinson’s disease. Proceedings of the National Academy of Science of the United States of America.

[CR7] Breska A, Ivry RB (2021). The human cerebellum is essential for modulating perceptual sensitivity based on temporal expectations. eLife.

[CR8] Callejas A, Shulman GL, Corbetta M (2014). Dorsal and ventral attention systems underlie social and symbolic cueing. Journal of Cognitive Neuroscience.

[CR9] Capizzi M, Correa Á, Vatakis A, Balci F, Di Luca M, Correa Á (2018). Measuring temporal preparation. Timing and time perception: Procedures, measures, and applications.

[CR10] Capizzi M, Correa A, Sanabria D (2013). Temporal orienting of attention is interfered by concurrent working memory updating. Neuropsychologia.

[CR11] Capizzi M, Correa Á, Wojtowicz A, Rafal RD (2015). Foreperiod priming in temporal preparation: Testing current models of sequential effects. Cognition.

[CR12] Capizzi M, Sanabria D, Correa Á (2012). Dissociating controlled from automatic processing in temporal preparation. Cognition.

[CR13] Chica AB, Bartolomeo P, Lupiáñez J (2013). Two cognitive and neural systems for endogenous and exogenous spatial attention. Behavioural Brain Research.

[CR14] Chica AB, Lupiáñez J, Bartolomeo P (2006). Dissociating inhibition of return from endogenous orienting of spatial attention: Evidence from detection and discrimination tasks. Cognitive Neuropsychology.

[CR15] Chica AB, Martín-Arévalo E, Botta F, Lupiáñez J (2014). The Spatial Orienting paradigm: how to design and interpret spatial attention experiments. Neuroscience and Biobehavioral Reviews.

[CR16] Correa A, Lupiáñez J, Madrid E, Tudela P (2006). Temporal attention enhances early visual processing: a review and new evidence from event-related potentials. Brain Research.

[CR17] Correa Á, Lupiáñez J, Milliken B, Tudela P (2004). Endogenous temporal orienting of attention in detection and discrimination tasks. Perception & Psychophysics.

[CR18] Correa A, Lupiáñez J, Tudela P (2005). Attentional preparation based on temporal expectancy modulates processing at the perceptual level. Psychonomic Bulletin & Review.

[CR19] Correa Á, Lupiáñez J, Tudela P (2006). The attentional mechanism of temporal orienting: determinants and attributes. Experimental Brain Research.

[CR20] Coull JT (2009). Neural substrates of mounting temporal expectation. PLoS Biology.

[CR21] Coull JT, Dehaene S, Brannon E (2011). Discrete neuroanatomical substrates for generating and updating temporal expectations. Time and Number in the Brain: Searching for the Foundations of Mathematical Thought.

[CR22] Coull JT, Nobre AC (1998). Where and when to pay attention: the neural systems for directing attention to spatial locations and to time intervals as revealed by both PET and fMRI. Journal of Neuroscience.

[CR23] Cravo AM, Rohenkohl G, Wyart V, Nobre AC (2013). Temporal expectation enhances contrast sensitivity by phase entrainment of low-frequency oscillations in visual cortex. Journal of Neuroscience.

[CR24] Cutanda D, Correa Á, Sanabria D (2015). Auditory temporal preparation induced by rhythmic cues during concurrent auditory working memory tasks. Journal of Experimental Psychology: Human Perception and Performance.

[CR25] Danziger S, Kingstone A, Snyder JJ (1998). Inhibition of return to successively stimulated locations in a sequential visual search paradigm. Journal of Experimental Psychology: Human Perception and Performance.

[CR26] Davranche K, Nazarian B, Vidal F, Coull J (2011). Orienting attention in time activates left intraparietal sulcus for both perceptual and motor task goals. Journal of Cognitive Neuroscience.

[CR27] De la Rosa MD, Sanabria D, Capizzi M, Correa Á (2012). Temporal preparation driven by rhythms is resistant to working memory interference. Frontiers in Psychology.

[CR28] Denison RN, Carrasco M, Heeger DJ (2021). A dynamic normalization model of temporal attention. Nature Human Behaviour.

[CR29] Doherty JR, Rao A, Mesulam MM, Nobre AC (2005). Synergistic effect of combined temporal and spatial expectations on visual attention. Journal of Neuroscience.

[CR30] Drazin DH (1961). Effects of foreperiod, foreperiod variability, and probability of stimulus occurrence on simple reaction time. Journal of Experimental Psychology.

[CR31] Duma GM, Granziol U, Mento G (2020). Should I stay or should I go? How local-global implicit temporal expectancy shapes proactive motor control: An hdEEG study. NeuroImage.

[CR32] Enns JT, Richards JC  (1997). Visual attentional orienting in developing hockey players. Journal of Experimental Child Psychology.

[CR33] Faugeras F, Naccache L (2016). Dissociating temporal attention from spatial attention and motor response preparation: A high-density EEG study. NeuroImage.

[CR34] Funes MJ, Lupiáñez J, Milliken B (2007). Separate mechanisms recruited by exogenous and endogenous spatial cues: Evidence from a spatial Stroop paradigm. Journal of Experimental Psychology: Human Perception and Performance.

[CR35] Gabay S, Henik A (2008). The effects of expectancy on inhibition of return. Cognition.

[CR36] Gabay S, Henik A (2010). Temporal expectancy modulates inhibition of return in a discrimination task. Psychonomic Bulletin & Review.

[CR37] Gibbon J (1977). Scalar expectancy theory and Weber’s law in animal timing. Pychological Review.

[CR38] Girardi G, Antonucci G, Nico D (2013). Cueing spatial attention through timing and probability. Cortex.

[CR39] Griffin IC, Miniussi C, Nobre AC (2002). Multiple mechanisms of selective attention: differential modulation of stimulus processing by attention to space or time. Neuropsychologia.

[CR40] Hayward D, Ristic J (2015). Exposing the cuing task: the case of gaze and arrow cues. Attention, Perception, & Psychophysics.

[CR41] Hayward DA, Ristic J (2016). Automated symbolic orienting is not modulated by explicit temporal attention. Acta Psychologica.

[CR42] Hein E, Rolke B, Ulrich R (2006). Visual attention and temporal discrimination: Differential effects of automatic and voluntary cueing. Visual Cognition.

[CR43] Herbst SK, Fiedler L, Obleser J (2018). Tracking temporal hazard in the human electroencephalogram using a forward encoding model. ENeuro.

[CR44] Janssen P, Shadlen MN (2005). A representation of the hazard rate of elapsed time in macaque area LIP. Nature Neuroscience.

[CR45] Jones A (2015). Independent effects of bottom-up temporal expectancy and top-down spatial attention: An audiovisual study using rhythmic cueing. Frontiers in Integrative Neuroscience.

[CR46] Jones A (2019). Temporal expectancies and rhythmic cueing in touch: The influence of spatial attention. Cognition.

[CR47] Jones A, Ward EV, Csiszer EL, Szymczak J (2022). Temporal expectation improves recognition memory for spatially attended objects. Journal of Cognitive Neuroscience.

[CR48] Jones MR, Moynihan H, MacKenzie N, Puente J (2002). Temporal aspects of stimulus-driven attending in dynamic arrays. Psychological Science.

[CR49] Jonides J, Long J, Baddeley A (1981). Voluntary versus automatic control over the mind’s eye’s movement. Attention and Performance XI.

[CR50] Kingstone A (1992). Combining expectancies. Quaterly Journal of Experimental Psychology.

[CR51] Kizuk SAD, Mathewson KE (2017). Power and phase of alpha oscillations reveal an interaction between spatial and temporal visual attention. Journal of Cognitive Neuroscience.

[CR52] Korolczuk I, Burle B, Coull JT (2018). The costs and benefits of temporal predictability: impaired inhibition of prepotent responses accompanies increased activation of task-relevant responses. Cognition.

[CR53] Kusnir F, Pesin S, Moscona G, Landau AN (2019). When temporal certainty doesn’t help. Journal of Cognitive Neuroscience.

[CR54] Langner R, Steinborn MB, Eickhoff SB, Huestegge L (2018). When specific action biases meet nonspecific preparation: Event repetition modulates the variable-foreperiod effect. Journal of Experimental Psychology: Human Perception and Performance.

[CR55] Large EW, Jones MR (1999). The dynamics of attending: How we track time varying events. Psychological Review.

[CR56] Lasaponara S, Chica AB, Lecce F, Lupiáñez J, Doricchi F (2011). ERP evidence for selective drop in attentional costs in uncertain environments: challenging a purely premotor account of covert orienting of attention. Neuropsychologia.

[CR57] Laidlaw KEW, Kingstone A (2017). If not when, then where? Ignoring temporal information eliminates reflexive but not volitional spatial orienting. Vision.

[CR58] Lupiáñez J, Martín-Arévalo E, Chica AB (2013). Is Inhibition of Return due to attentional disengagement or to a detection cost? The detection cost theory of IOR. Psicológica.

[CR59] Lupiáñez J, Milán EG, Tornay FJ, Madrid E, Tudela P (1997). Does IOR occur in discrimination tasks? Yes, it does, but later. Perception & Psychophysics.

[CR60] MacKay A, Juola JF (2007). Are spatial and temporal attention independent?. Perception & Psychophysics.

[CR61] Marzecová A, Schettino A, Widmann A, SanMiguel I, Kotz SA, Schröger E (2018). Attentional gain is modulated by probabilistic feature expectations in a spatial cueing task: ERP evidence. Scientific Reports.

[CR62] Mathewson KE, Fabiani M, Gratton G, Beck DM, Lleras A (2010). Rescuing stimuli from invisibility: Inducing a momentary release from visual masking with pre-target entrainment. Cognition.

[CR63] Mattes S, Ulrich R (1997). Response force is sensitive to the temporal uncertainty of response stimuli. Perception & Psychophysics.

[CR64] McCormick CR, Redden RS, Lawrence MA, Klein RM (2018). The independence of endogenous and exogenous temporal attention. Attention, Perception & Psychophysics.

[CR65] Milliken B, Lupiáñez J, Roberts M, Stevanovski B (2003). Orienting in space and time: Joint contributions to exogenous spatial cuing effects. Psychonomic Bulletin & Review.

[CR66] Miniussi C, Wilding EL, Coull JT, Nobre AC (1999). Orienting attention in time. Modulation of brain potentials. Brain.

[CR67] Näätänen R (1972). Time uncertainty and occurrence uncertainty of the stimulus in a simple reaction time task. Acta Psychologica.

[CR68] Ni AM, Maunsell JHR (2019). Neuronal Effects of Spatial and Feature Attention Differ Due to Normalization. The Journal of Neuroscience.

[CR69] Niemi P, Näätänen R (1981). Foreperiod and simple reaction time. Psychological Bulletin.

[CR70] Nobre AC, Coull JT, Nobre AC (2010). How can temporal expectations bias perception and action. Attention and Time.

[CR71] Nobre AC, van Ede F (2018). Anticipated moments: temporal structure in attention. Nature Reviews. Neuroscience.

[CR72] Olk B (2014). Effects of spatial, temporal and spatiotemporal cueing are alike when attention is directed voluntarily. Experimental Brain Research.

[CR73] O’Reilly JX, Schuffelgen U, Cuell SF, Behrens TE, Mars RB, Rushworth MF (2013). Dissociable effects of surprise and model update in parietal and anterior cingulate cortex. Proceedings of the National Academy of Sciences, USA.

[CR74] Posner MI (1980). Orienting of attention. Quaterly Journal of Experimental Psychology.

[CR75] Posner MI, Nissen M, Odgen W, Pick HL, Saltzman E (1978). Attended and unattended processing modes: the role of set for spatial location. Modes of Perceiving and Processing Information.

[CR76] Posner MI, Petersen SE (1990). The attention system of the human brain. Annual Review of Neuroscience.

[CR77] Posner MI, Rafal RD, Choate LS, Vaughan J (1985). Inhibition of return: Neural basis and function. Cognitive Neuropsychology.

[CR78] Rimmele J, Jolsvai H, Sussman E (2011). Auditory target detection is affected by implicit temporal and spatial expectations. Journal of Cognitive Neuroscience.

[CR79] Ristic J, Kingstone A (2012). A new form of human spatial attention: Automated symbolic orienting. Visual Cognition.

[CR80] Rohenkohl G, Coull JT, Nobre AC (2011). Behavioural dissociation between exogenous and endogenous temporal orienting of attention. PLoS ONE.

[CR81] Rohenkohl G, Cravo AM, Wyart V, Nobre AC (2012). Temporal expectation improves the quality of sensory information. The Journal of Neuroscience.

[CR82] Rohenkohl G, Gould IC, Pessoa J, Nobre AC (2014). Combining spatial and temporal expectations to improve visual perception. Journal of Vision.

[CR83] Rolke B, Festl F, Seibold VC (2016). Toward the influence of temporal attention on the selection of targets in a visual search task: An ERP study. Psychophysiology.

[CR84] Sanabria D, Capizzi M, Correa Á (2011). Rhythms that speed you up. Journal of Experimental Psychology: Human Perception and Performance.

[CR85] Sani I, Stemmann H, Caron B (2021). The human endogenous attentional control network includes a ventro-temporal cortical node. Nature Communications.

[CR86] Seibold VC, Balke J, Rolke B (2023). Temporal attention. Frontiers in Cognition.

[CR87] Seibold VC, Stepper MY, Rolke B (2020). Temporal attention boosts perceptual effects of spatial attention and feature-based attention. Brain and Cognition.

[CR88] Sharp P, Melcher D, Hickey C (2019). Different effects of spatial and temporal attention on the integration and segregation of stimuli in time. Attention, Perception, & Psychophysics.

[CR89] Tal-Perry N, Yuval-Greenberg S (2022). The spatiotemporal link of temporal expectations: Contextual temporal Expectation is independent of spatial attention. The Journal of Neuroscience.

[CR90] Tang X, Li C, Li Q, Gao Y, Yang W, Yang J, Ishikawa S, Wu J (2013). Modulation of auditory stimulus processing by visual spatial or temporal cue: an event-related potentials study. Neuroscience Letters.

[CR91] Tassinari G, Berlucchi G (1993). Sensory and attentional components of slowing of manual reaction time to non-fixated visual targets by ipsilateral primes. Vision Research.

[CR92] Tassinari G, Aglioti S, Chelazzi L, Peru A, Berlucchi G (1994). Do peripheral non-informative cues induce early facilitation of target detection?. Vision Research.

[CR93] Taylor TL, Klein R (2000). Visual and motor effects in inhibition of return. Journal of Experimental Psychology: Human Perception and Performance.

[CR94] Tipper C, Kingstone A (2005). Is inhibition of return a reflexive effect?. Cognition.

[CR95] Trillenberg P, Verleger R, Wascher E, Wauschkuhn B, Wessel K (2000). CNV and temporal uncertainty with “ageing” and “non-ageing” S1–S2 intervals. Clinical Neurophysiology.

[CR96] Triviño M, Arnedo M, Lupiáñez J, Chirivella J, Correa A (2011). Rhythms can overcome temporal orienting deficit after right frontal damage. Neuropsychologia.

[CR97] Vallesi A, Lozano V, Correa A (2013). Dissociating temporal preparation processes as a function of the inter-trial interval duration. Cognition.

[CR98] Vangkilde S, Coull JT, Bundesen C (2012). Great expectations: temporal expectation modulates perceptual processing speed. Journal of Experimental Psychology Human Perception and Performance.

[CR99] Visalli A, Capizzi M, Ambrosini E, Mazzonetto I, Vallesi A (2019). Bayesian modeling of temporal expectations in the human brain. NeuroImage.

[CR100] Visalli A, Capizzi M, Ambrosini E, Kopp B, Vallesi A (2021). Electroencephalographic correlates of temporal Bayesian belief updating and surprise. NeuroImage.

[CR101] Visalli A., Capizzi M., Ambrosini E., Kopp B., & Vallesi, A. (2023) P3-like signatures of temporal predictions: a computational EEG study. *Experimental Brain Research*. 10.1007/s00221-023-06656-z 10.1007/s00221-023-06656-z 37354350

[CR102] Vossel S, Mathys C, Daunizeau J, Bauer M, Driver J, Friston KJ, Stephan KE (2014). Spatial attention, precision, and Bayesian inference: A study of saccadic response speed. Cerebral Cortex.

[CR103] Weinbach N, Henik A (2012). Temporal orienting and alerting - the same or different?. Frontiers in Psychology.

[CR104] Weinbach N, Shofty I, Gabay S, Henik A (2015). Endogenous temporal and spatial orienting: Evidence for two distinct attentional mechanisms. Psychonomic Bulletin & Review.

[CR105] Wilsch A, Mercier MR, Obleser J, Schroeder CE, Haegens S (2020). Spatial attention and temporal expectation exert differential effects on visual and auditory discrimination. Journal of Cognitive Neuroscience.

[CR106] Wolfe JM (2021). Guided Search 6.0: An updated model of visual search. Psychonomic Bulletin & Review.

